# Impact of COVID-19 pandemic in adolescents on HIV treatment

**DOI:** 10.11604/pamj.2022.42.120.33774

**Published:** 2022-06-14

**Authors:** Brian Van Wyk, Yolanda Mayman

**Affiliations:** 1 School of Public Health, University of the Western Cape, Bellville, South Africa

**Keywords:** Adolescents, HIV, antiretroviral therapy, COVID-19

## Abstract

The COVID-19 pandemic and concomitant lockdown restrictions in South Africa pose critical challenges for adolescents living with HIV (ALHIV) and on ART, impacting their ability to remain engaged in care and adherent to antiretroviral therapy (ART). Not only has this pandemic directly impacted the health care that ALHIV receive, but it has also consequently had devastating effects on society and has negatively affected the mental health and well-being of ALHIV. The challenges associated with the impact of the COVID-19 pandemic on disadvantaged groups such as ALHIV on ART need to be further explored as it may offer hope to ALHIV and restore confidence in the health system.

## Commentary

The COVID-19 pandemic brought about severe economic and social inequalities that continue to negatively impact the lives and experiences of already disadvantaged groups such as adolescents living with HIV (ALHIV) [1]. Further disruptions to the economy, social contexts and health services caused by the pandemic negatively affects both psychosocial and clinical outcomes [2]. This commentary highlights the impact that the COVID-19 pandemic has on adolescents living with HIV (ALHIV) and HIV treatment. An advocacy brief on children states the impact of the coronavirus extends across every dimension of the lives of children and their well-being. We comment on how national responses to combat the COVID-19 pandemic has led to the disruption of health services and the care of children, negatively affected nutrition and food security of children, and had detrimental effects their mental health and wellbeing [3].

### COVID-19 and ALHIV

Sub-saharan Africa houses the largest population of ALHIV as 89% of the 1.6 million ALHIV globally reside here [4]. It has been found that the HIV epidemic disproportionately affects groups from communities heavily impacted by social determinants of poor health including poverty, unemployment, discrimination and stigma [1]. It has been noted that adolescents and young adults living with HIV (10-24 years) are the most vulnerable and experience poor treatment outcomes across every phase the of HIV care continuum (testing, diagnosis, adherence to medication, viral suppression) due to health and socioeconomic inequalities [1,2,4]. This group also experiences a higher rate of loss to follow-up, virological failure, and mortality in comparison to adults on antiretroviral therapy (ART) [2]. Not only do these inequalities negatively impact the health of adolescents and young adults living with HIV, but it also increases their risk for and susceptibility to HIV transmission within their groups and communities [1].

### ALHIV and health services

Social and health service delivery restrictions caused by national responses to curb the spread of COVID-19 disrupted and hindered access to facility-based services such as medication pick-ups, adherence counselling and support groups [2]. The loss of household income and restrictions on public transport limited the ability of ALHIV to access health services [5]. It has been previously reported that ALHIV in sub-Saharan Africa are at high risk of anxiety, depression and interpersonal violence, and these conditions are exacerbated due to lockdown restrictions and prolonged isolation at home. In the time(s) of lockdowns, ALHIV faced structural challenges such as the unavailability of safe centres for health and-, mental health issues and social care, in the presence of required parental consent which further restrict their access to health care and interrupt ART adherence [6]. The COVID-19 pandemic impacted the informal economy greatly; which increased unemployment and income generation and led to widespread food insecurity [7]. For ALHIV who attend school, schools not only provides a space for freedom, but also allows for peer interaction and psychological support. Furthermore, in Sub-Saharan Africa the effects of COVID-19 on ALHIV may be especially detrimental due to the time, effort and resources that ALHIV have to put into seeking and obtaining care [4]. This burden is further exacerbated by the limited availability of HIV services, as well as ALHIV´s struggle in having to mitigate social stigma regarding treatment [4]. ALHIV face a wide range of challenges as they have to live with a chronic condition and the concomitant stigmatisation, while simultaneously dealing with normal adolescent development [8]. Most often these stigmas are negative in nature and can invoke feelings of shame, helplessness and worthlessness within individuals living with HIV [9]. All of these factors are known to negatively impact the mental health and psychological well-being of ALHIV.

### ALHIV and mental health

The impact of the COVID-19 pandemic on the mental health of children can be noted in social isolation, the loss of loved ones, the disruption of school programmes as well as food insecurity all directly and in increased levels of stress in households [10]. The responses to these stressors may appear as new patterns of behaviour which reflect coping mechanisms, such as sleep problems, an increase in clingy behaviours and anxiety or pre-occupation with rituals relating to health and wellness [10]. Research shows that the mental health conditions and state among ALHIV are often associated with negative treatment outcomes [11]. It is further argued that individuals with HIV may experience a stronger stress response in comparison to the rest of the population, due to their increased risk of contracting COVID-19 [12,13]. ALHIV still experience higher risk of facing various illness-related stressors such as dealing with social stigma, secrecy regarding their status, feelings of isolation, issues of disclosure of their HIV status [14,15].

Adolescents are in the critical stages of biological, psychological, behavioural, cognitive and social development, and it is found that adolescents greatly benefit from and place great value on social interaction and in-person peer contact [6]. However, the COVID-19 pandemic and strategies such as the closing of schools, lockdowns, isolation and quarantine, implemented to prevent the spreading of the coronavirus, are said to result in poorer mental health among adolescents [6]. Researchers also mention that there is a lack of data on the impact and consequences of the COVID-19 pandemic on healthcare systems and in particular the response to controlling the HIV epidemic [16]. It is therefore necessary that research be undertaken to further explore the impact of the COVID-19 global pandemic on the ART adherence of ALHIV. This can assist in the development of programmes aimed at supporting ALHIV during this pandemic.
